# Dose calculation differences between Monte Carlo and pencil beam depend on the tumor locations and volumes for lung stereotactic body radiation therapy

**DOI:** 10.1120/jacmp.v14i2.4011

**Published:** 2013-03-04

**Authors:** Tingliang Zhuang, Toufik Djemil, Peng Qi, Anthony Magnelli, Kevin Stephans, Gregory Videtic, Ping Xia

**Affiliations:** ^1^ Department of Radiation Oncology Taussig Cancer Center, Cleveland Clinic Cleveland OH USA

**Keywords:** lung SBRT, dose calculation, Monte Carlo, pencil beam

## Abstract

Stereotactic body radiation therapy (SBRT) has been increasingly used as an efficacious treatment modality for early‐stage non‐small cell lung cancer. The accuracy of dose calculations is compromised due to the presence of inhomogeneity. For the purpose of a consistent prescription, radiation doses were calculated without heterogeneity correction in several RTOG trials. For patients participating in these trials, recalculations of the planned doses with more accurate dose methods could provide better correlations between the treatment outcomes and the planned doses. Using a Monte Carlo (MC) dose calculation algorithm as a gold standard, we compared the recalculated doses with the MC algorithm to the original pencil beam (PB) calculations for our institutional clinical lung SBRT plans. The focus of this comparison is to investigate the volume and location dependence on the differences between the two dose calculations. Thirty‐one clinical plans that followed RTOG and other protocol guidelines were retrospectively investigated in this study. Dosimetric parameters, such as $D_{1}$, $D_{95}$, and $D_{99}$ for the PTV and $D_{1}$ for organs at risk, were compared between two calculations. Correlations of mean lung dose and $V_{20}$ of lungs between two calculations were investigated. Significant dependence on tumor size and location was observed from the comparisons between the two dose calculation methods. When comparing the PB calculations without heterogeneity correction to the MC calculations with heterogeneity correction, we found that in terms of $D_{95}$ of PTV: (1) the two calculations resulted in similar $D_{95}$ for edge tumors with volumes greater than 25.1 cc; (2) an average overestimation of 5% in PB calculations for edge tumors with volumes less than 25.1 cc; and (3) an average overestimation of 9% or underestimation of 3% in PB calculations for island tumors with volumes smaller or greater than 22.6 cc, respectively. With heterogeneity correction, the PB calculations resulted in an average reduction of 23.8% and 15.3% in the $D_{95}$ for the PTV for island and edge lesions, respectively, when compared to the MC calculations. For organs at risks, very small differences were found among all the comparisons. Excellent correlations for mean dose and $V_{20}$ of lungs were observed between the two calculations. This study demonstrated that using a single scaling factor may be overly simplified when accounting for the effects of heterogeneity correction. Accurate dose calculations, such as the Monte Carlo algorithms, are highly recommended to understand dose responses in lung SBRT.

PACS number: 87.53.Ly

## I. INTRODUCTION

Stereotactic body radiation therapy (SBRT) has been increasingly used in managing non‐small cell lung cancer (NSCLC) as a noninvasive alternative to surgery. Clinical trials have demonstrated efficacy of this treatment modality with excellent local control rate and tolerable normal tissue toxicity.^(^ ^1^ ^–^ ^5^ ^)^

The precision of radiation dose delivery of SBRT is ensured by using extra imaging systems in the treatment room for patient positioning and by using treatment machines that have high mechanical and dosimetric accuracy. However, the knowledge regarding the actual dose delivered to the tumor is not satisfactory. One reason is the tumor motion during treatment, although this may be controlled by using advanced techniques to manage respiratory motion.^(^ ^6^ ^)^ Another challenge comes from the dose calculation in a heterogeneous medium.^(^ ^7^ ^)^ Currently, dose calculation algorithms with heterogeneity correction are not consistent among different treatment planning systems,^(^ ^8^ ^–^ ^12^ ^)^ depending on how the changes in lateral electron transport are taken into account.^(^ ^13^ ^)^ For this reason, dose calculations without heterogeneity correction were required in RTOG 0236,^(^ ^14^ ^)^ which was a phase II trial of SBRT in the early stages of medically inoperable NSCLC. This requirement is supported by the consistent performance of dose calculation algorithms in different treatment planning systems for homogeneous water. However, to better understand the correlation between dose fractionation schemes and treatment outcomes, accurate dose calculations with heterogeneity correction are highly desired. One such dose calculation algorithm is the collapsed cone convolution algorithm implemented in the Pinnacle treatment planning system (Philips Healthcare, Andover, MA), which has demonstrated accurate dose calculations in the heterogeneous medium.^(^ ^11^ ^,^ ^12^ ^)^ Using the Pinnacle treatment planning system (TPS), Xiao et al.^(^ ^15^ ^)^ have retrospectively analyzed treatment plans submitted to RTOG 0236 from multiple institutions. Significant dose differences were found when heterogeneity correction was applied. To account for the differences, a suggestion was made to rescale the prescription from 60 Gy to 54 Gy. However, a single scaling factor may be overly simplified because the differences in dose calculations with and without heterogeneity correction may be dependent on tumor locations and volumes.

The aim of the current study was to investigate the possible tumor size and location dependence of actual planned doses for lung SBRT cases using a Monte Carlo (MC) dose calculation algorithm compared to clinically used pencil beam calculations. Since the Monte Carlo algorithm calculates doses by simulating the interactions between photons and matter, it should, in principle, give more accurate doses. Other studies have shown that the superposition/convolution algorithm such as collapsed cone convolution algorithm in the Pinnacle TPS has similar performances as the MC for dose calculations in heterogeneous mediums.^(^ ^9^ ^)^ In this study, the MC algorithm was considered as the gold standard for dose calculations. Both the MC and PB dose calculation algorithms were implemented in the iPlan RT 4.1.2 TPS (BrainLAB AG, Feldkirchen, Germany). The improvement in dose calculation accuracy by using the MC algorithm in the iPlan TPS over other algorithms has been demonstrated by several investigators.^(^ ^16^ ^–^ ^18^ ^)^ Excellent agreements between MC calculations and measurements have been reported for both homogeneous and heterogeneous phantoms. These phantom studies have shown that, for small targets imbedded in low‐density environments, the MC calculated doses were significantly different from those calculated with the PB algorithm. Therefore, we are aiming to (1) obtain the planned doses using MC calculations for lung cancer patients treated with SBRT where the PB algorithms were used, and (2) study the possible dependence on tumor volumes and locations when comparing the MC and PB calculations. For these purposes, we presented comparisons between clinical plans that were calculated by using PB without heterogeneity correction to MC calculations with heterogeneity correction. We also presented comparisons of dose calculated with the MC and PB with or without heterogeneity correction.

## II. MATERIALS AND METHODS

### A. Dose calculation algorithms in iPlan RT 4.1.2 TPS

Two types of dose calculation algorithms, pencil beam and Monte Carlo, have been implemented in BrainLAB iPlan RT 4.1.2 TPS. The MC dose calculation algorithm in iPlan RT 4.1.2 TPS was based on the X‐ray Voxel Monte Carlo algorithm.^(^ ^19^ ^)^ This algorithm consists of three main components: source modeling, beam collimating system modeling, and patient dose computation. Depending on the desired precision and calculation speed, there are two options for MLC modeling which are “accuracy optimized” and “speed optimized”. There are four parameters for dose calculations in iPlan: spatial resolution, mean variance, dose result type, and MLC model. The spatial resolution defines the size of the dose calculation grid. The mean variance estimates the statistical uncertainty of the MC dose calculation. Using a smaller variance, the dose calculations would be more accurate. However, the computation time would be longer. The dose result type can be chosen from “dose to water” or “dose to medium”. The parameter was set to be “dose to medium” in all the calculations in this paper. The MLC model parameter has been described above. (Readers can refer to the BrainLAB Technical Reference Guide for more details.)

### B. Verification of Monte Carlo algorithm in water

Verification of MC dose calculations in both homogeneous and heterogeneous medium in iPlan RT Dose 4.1.2 have been reported by several researchers. Results showed the calculated doses agreed with the measured ones within 2% in high‐dose regions and 2 mm in high‐gradient regions,^(^ ^17^ ^,^ ^18^ ^)^ and the averaged one‐dimensional gamma values did not exceed 0.3 with 2%/2 mm criterion when comparing calculated and measured dose distributions.^(^ ^16^ ^)^ The purpose of this paper is to reevaluate the treatment plans calculated with the pencil beam algorithm by comparing with Monte Carlo calculations. To access the accuracy for squared fields, we presented comparisons of percentage depth dose (PDD), profiles, and output factors measured at 100 SSD in a uniform water phantom to MC calculations. A 2 mm dose grid, 1.5% variance, and “accuracy optimized” for MLC modeling were used for MC dose calculations.

### C. Patient selection, treatment planning, and dose calculation

**Figure 1:**
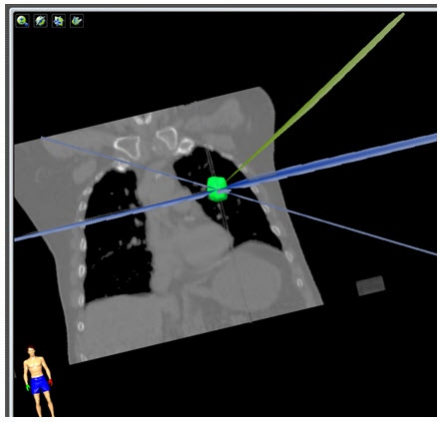
Demonstration of a dynamic conformal arc plan for a lung SBRT treatment.

Patient data were retrospectively collected from an institutional review board approved registry. Patients were treated with SBRT in Novalis (BrainLAB, Feldkirchen, Germany) platform for a total dose of 60 Gy in three fractions^(^ ^14^ ^)^ or 30 Gy in a single fraction (www.ClinicalTrials.gov identifier: NCT00843726, a phase III trial led by Roswell Park Cancer Institute, Buffalo, NY). Treatment plans were designed using dynamic conformal arcs with 6 MV photon beams in iPlan RT 4.1.2 TPS following the protocol guidelines. Each clinical plan was normalized such that 95% of the planning target volume (PTV) received the prescription dose. We used partial arcs at table angles of 0°, 30°, and 330°, as shown in Fig. 1. The deliverable angular range of each arc was determined by performing a “dry run” prior to the treatment in order to avoid collisions.

According to these two protocols (RTOG 0236 and Roswell Park), the clinical plans were calculated using the pencil beam algorithm without heterogeneity correction. To evaluate the impact of using a more sophisticated dose calculation algorithm (such as MC) and heterogeneity correction, four additional plans were generated by recalculating the clinical plan. Different combinations of dose algorithms and heterogeneity correction were used in these calculations, as listed in Table 1. The plan labeled “PBHomo” was the original clinical plan. The recalculations of this plan using MC with and without heterogeneity correction were labeled as “MCHete*” and “MCHomo”, respectively. These three plans had the same MU settings. The plan labeled as “PBHete” was calculated by applying heterogeneity correction to the “PBHomo” and renormalized to have the same coverage. Recalculation of “PBHete” with MC was labeled as “MCHete” which had the same MU setting as “PBHete”. The dose grid for all the calculations was 2 mm. The “accuracy optimized” for MLC modeling was selected for MC calculation. A 2% variance (instead of 1.5% for PDDs and profiles calculations) was used in patient plan calculations so that the total dose calculation time would be several minutes. In the overlapping region, note the variance was smaller. Therefore, dose calculation uncertainty in the PTV was less than 2%.

**Table 1:** Summary of the five calculations.

*Plan Name*	*Dose Calculation Algorithm*	*Heterogeneity Correction*	*MU*
PBHomo	PB	No	${\text{MU}}_{1}$
MCHomo	MC	No	${\text{MU}}_{1}$
MCHete^*^	MC	Yes	${\text{MU}}_{1}$
PBHete	PB	Yes	${\text{MU}}_{2}$
MCHete	MC	Yes	${\text{MU}}_{2}$

### D. Data analysis

The five calculations described in the Material and Methods Section C above were exported in DICOM format. The dose and volume data for PTV and organs at risk (OAR) listed in Table 2 were extracted from the DICOM files for the five plans, respectively, using the in‐house MATLAB (The MathWorks, Natick, MA) code and the computational environment for radiotherapy research (CERR)^(^ ^20^ ^)^ software.

**Table 2:** Dose and volume indices for the five plans.

*Regions of interest*	*Parameters*
PTV	$D_{1}$, $D_{95}$, $D_{99}$
Total lung	Mean lung dose (MLD), $V_{20}$
Other organs at risk	$D_{1}$

Based on parameters listed in Table 2, the following metrics were derived to evaluate the differences and relations among the five calculations:

- $R{\left(D_{x}\right)}^{2,1}$: Ratio of $D_{x}$ of PTV between two calculations defined as $\frac{D_{x}^{plan2}}{D_{x}^{plan1}}$, where $D_{x}$ represents the dose to *x* percent of the PTV and *x* was 1%, 95%, or 99%. We compared the quantity $\left[R{\left(D_{x}\right)}^{2,1}-1\right]$ which represented the percentage difference between two calculations;
- Percentage of cases in which the difference in $D_{95}$ of PTV between two calculations was more than 7%. The 7% dose difference was chosen since it might be detectable from clinical outcomes;^(^ ^21^ ^)^ and
- Correlation of mean lung dose (MLD) and $V_{20}$ of lungs between two calculations.

Comparisons between MCHete^*^ and PBHomo were of great interest since the calculations with MC and heterogeneity correction in MCHete^*^ gave the actual planned doses, which may have been different from the dose given by PB calculations without heterogeneity correction.

**Figure 2:**
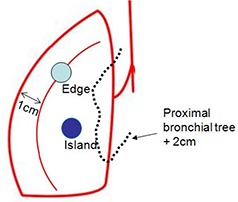
Illustration of the locations for edge and island tumors.

To study the tumor location dependence of the dose differences between MC and PB calculations, all lesions were separately grouped based on the distance between the GTV contours to the chest wall. A lesion was considered as an edge case if the distance was smaller than 1 cm; otherwise, it was considered as an island lesion, as shown in Fig. 2. The comparisons were conducted separately for the two types of lesions.^†^

## III. RESULTS

### A. Verification of Monte Carlo algorithm in water

**Figure 3:**
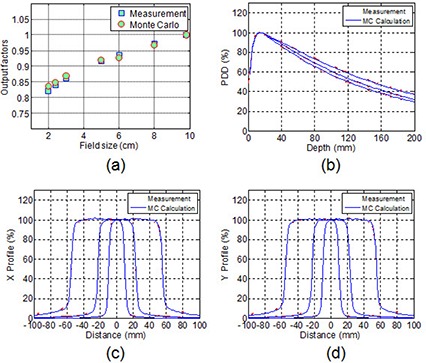
Comparison of output factors (a), and percentage depth dose (b), and beam profiles (c) and (d) for square fields of 1.8×1.8×,4.2×4.2, and 10×10 between the MC calculations and measurements in water phantom.

Good agreement between the MC calculated and the measured PDDs and profiles for field sizes of 1.8×1.8×4.2×4.2, and $9.8\times 9.8\;{\text{cm}}^{2}$, and output factors for field sizes of 2×2,2.4×2.4×3×3,5×5,6×6,8×8, and $9.8\times 9.8\;{\text{cm}}^{2}$ in water are shown in Fig. 3. The averaged percentage difference was 0.57±0.28 for all PDDs. The average percentage difference was 0.61±0.72 for profiles in low‐dose gradient regions, and the average distance to agreement in penumbra regions was 0.28 mm. The average percentage difference was 0.90±0.62 for the output factors.

### B. Patient plan calculation comparisons

#### B.1 Patient statistics

Thirty‐one clinical plans were recalculated for this study. The PTV volume ranged from 8.4 cc to 83.3 cc, with a mean volume of 28.2 cc. Among these patients, 16 cases were island and 15 cases were edge lesions.

#### B.2 Without heterogeneity correction

Table 3 lists comparisons of various dose and volume endpoints for MC and PB calculations without heterogeneity correction for all patients. Very small differences were observed for these parameters.

**Table 3:** Comparisons between plans PBHomo and MCHomo.

*Parameters*	*All Cases*
$\left[R{\left(D_{1}\right)}^{MC,PB}-1\right]\left(\%\right)$	−0.01±0.6
$\left[R{\left(D_{95}\right)}^{MC,PB}-1\right]\left(\%\right)$	0.6±0.7
$\left[R{\left(D_{99}\right)}^{MC,PB}-1\right]\left(\%\right)$	0.7±0.9
$\left[{\text{MLD}}_{\text{MC}}-{\text{MLD}}_{\text{PB}}\right]\left(\text{Gy}\right)$	0.0±0.06
$\left[V_{20,\text{MC}}-V_{20,\text{PB}}\right]\left(\%\right)$	0.1±0.1
$\left[D_{1,\text{MC}}-D_{1,\text{PB}}\right]\;\text{of}\;\text{Cord}(\text{Gy})$	0.06±0.2
$\left[D_{1,\text{MC}}-D_{1,\text{PB}}\right]\;\text{of}\;\text{Esophagus}(\text{Gy})$	0.07±0.2
$\left[D_{1,\text{MC}}-D_{1,\text{PB}}\right]\;\text{of}\;\text{Brachial}\;\text{Plexus}\;(\text{Gy})$	0.08±0.6

**Figure 4:**
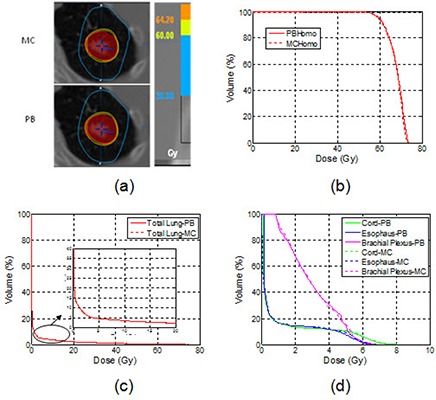
An example of the axial isodose distributions (a), DVHs of the PTV (b), DVHs of the lungs (c), and other OARs (d) for plans calculated with the MC and the PB without heterogeneity correction.

Figure 4 shows isodose distributions and DVHs from PB and MC calculations without heterogeneity correction, respectively, in a selected case with PTV volume of 16.9 cc in the left upper lobe. Both isodose lines and DVHs of PTV and OARs from the two calculations were in good agreement.

This comparison demonstrated good agreement between MC and PB dose calculations in uniform water regardless of tumor sizes and locations.

#### B.3 With heterogeneity correction

Table 4 shows selected endpoints for MC and PB calculations with heterogeneity correction. When compared to PB calculations, MC calculations resulted in an average reduction of 11.6%/7.5% in $D_{1}$, 23.8%/15.3% in $D_{95}$, and 25.2%/16.7% in $D_{99}$ for island and edge tumors, respectively.

**Table 4:** Comparisons between plans PBHete and MCHete.

*Parameters*	*Island Lesions (n*=*16)*	*Edge Lesions (n*=*15)*
$\left[R{\left(D_{1}\right)}^{MC,PB}-1\right]\left(\%\right)$	−11.6±2.7	−7.5±3.1
$\left[R{\left(D_{95}\right)}^{MC,PB}-1\right]\left(\%\right)$	−23.8±7.6	−15.3±6.5
$\left[R{\left(D_{99}\right)}^{MC,PB}-1\right]\left(\%\right)$	−25.2±8.1	−16.7±6.9
$\left[{\text{MLD}}_{\text{MC}}-{\text{MLD}}_{\text{PB}}\right]\left(\text{Gy}\right)$	−0.1±0.1	−0.2±0.07
$\left[V_{20,\text{MC}}-V_{20,\text{PB}}\right]\left(\%\right)$	−0.3±0.2	−0.2±0.1
$\left[D_{1,\text{MC}}-D_{1,\text{PB}}\right]\;\text{of}\;\text{Cord}\;(\text{Gy})$	−0.2±0.2	−0.3±0.2
$\left[D_{1,\text{MC}}-D_{1,\text{PB}}\right]\;\text{of}\;\text{Esophagus}\;(\text{Gy})$	−0.3±0.1	−0.4±0.3
$\left[D_{1,\text{MC}}-D_{1,\text{PB}}\right]\;\text{of}\;\text{Brachial}\;\text{Plexus}\;(\text{Gy})$	−0.04±0.1	−0.1±0.3

**Figure 5:**
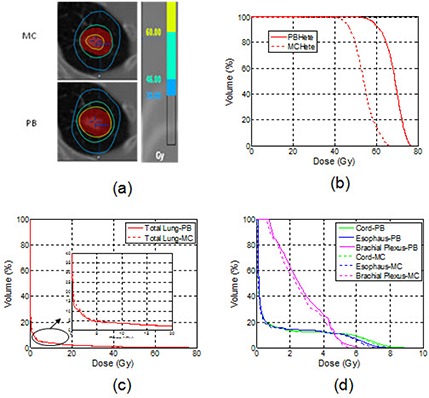
An example of the axial isodose distributions (a), DVHs of the PTV (b), DVHs of the lungs (c), and other OARs (d) for plans calculated with the MC and the PB with heterogeneity correction.

For the same patient plan used in Fig. 4, the isodose lines and DVHs using PB and MC calculations with heterogeneity correction are shown in Fig. 5. In the clinical plan with the PB calculation, 95% PTV was adequately covered by the prescription (60 Gy) isodose line. With MC recalculation, $D_{95}$ was reduced from 60 Gy to 46 Gy, indicating a 30% dose overestimation by the PB calculation. As shown in Fig. 5(b), there was a large difference between two DVHs of PTV with the PB and MC calculation.

The differences in $D_{95}$ of the PTV between MC and PB calculations were greater than 7% of the prescription dose in 100% of island lesions, 86.7% of edge lesions, and 93.5% of all lesions.

**Figure 6:**
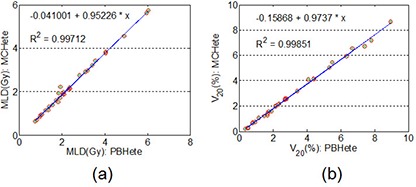
Correlations for the MLD (a) and the $V_{20}$ (b) of lungs for plans calculated with the MC and the PB with heterogeneity correction.

From Table 4, the differences in $D_{1}$ of OARs were negligible between MC and PB calculations for both edge and island cases. Also, as shown in Figs. 5(c) and (d), small but clinically insignificant differences were observed in the low‐dose region for the OARs listed. For MLD and $V_{20}$ of lungs, we found excellent correlations between PB and MC calculations, as shown in Fig. 6. A linear fitting results in straight lines with slopes of 0.95 and 0.97 for MLD and $V_{20}$ of lungs, respectively. This result demonstrated that without heterogeneity correction, the PB calculation would result in 5% overestimation of MLD and 3% overestimation of the $V_{20}$ of lungs when compared to the MC calculation.

#### B.4 Pencil beam without heterogeneity compared to Monte Carlo with heterogeneity correction

**Figure 7:**
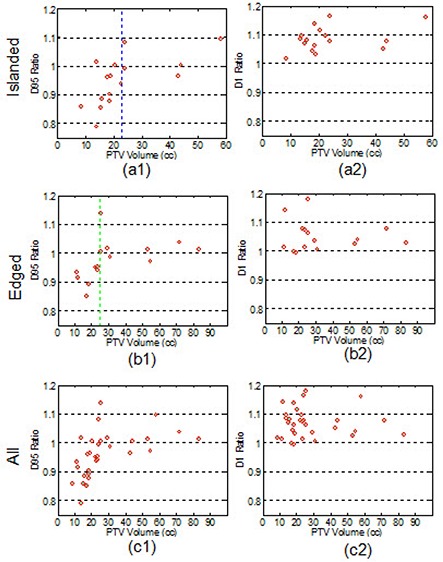
Differences in $D_{95}$ (a1)–(c1) and $D_{1}$ (a2)–(c2) between the PB calculations without heterogeneity correction and the MC calculations with heterogeneity correction.

In (Figs. 7a1) to 7(c1), the ratios of $D_{95}$ between the plans MCHete^*^ and PBHomo were plotted verses the target volumes for island, edge, and all tumors. The corresponding plots for $D_{1}$ of the PTV are presented in (Figs. 7a2) to 7(c2).

In 50% of island cases, 26.7% of edge lesions, and 38.7% among all cases, the dose differences in $D_{95}$ between MC calculations and PB calculations were more than 7% of the prescriptions. Regarding the hot spot in the plans, we noticed the ratio of $D_{1}$ between the two calculations was greater than 1 in 93.6% of all patients, which indicates that MC calculations generally resulted in “hotter” plans.

Based on the results shown in Fig. 7, we found that the ratio of $D_{95}$ between the two calculations was 0.95±0.08 and 1.01±0.02 for edge tumors with volumes smaller and larger than 25.1 cc (indicated by the green line in (Fig. 7b1)), respectively. For island tumors, the ratio of $D_{95}$ was 0.91±0.07 and 1.03±0.06 for volume smaller and larger than 22.6 cc, respectively (indicated by the blue line in (Fig. 7a1)).

**Figure 8:**
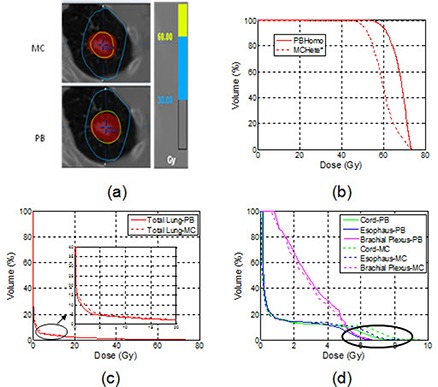
An example of the axial isodose distributions (a), DVHs of the PTV (b), DVHs of the lungs(c), and other OARs (d) for the MC calculations with heterogeneity correction and the PB without heterogeneity correction.

For the same patient plan used in Fig. 4, the differences in isodose lines and DVHs for the two calculations are shown in Fig. 8. In this case the 60 Gy isodose line failed to cover 95% of PTV in MC calculation.

Table 5 shows the comparison between the MC and PB calculations for OARs. The differences of MLD and $V_{20}$ for lungs between the two calculations were similar for island and edge lesions. For other OARs farther away from the target (such as brachial plexus in island lesion cases), the differences in $D_{1}$ between MC and PB calculations were also negligible. As shown in Fig. 8(c), the PB calculation slightly underestimated in the low‐dose region of the lung DVH compared to the MC calculation. Noticeable, but not clinically significant, differences between the two calculations existed for OARs that were close to the target (e.g., spinal cord) as shown by the highlighted circle in the DVH comparisons in Fig. 8(d).

**Table 5:** Comparisons between plans PBHomo and MCHete^*^

*Parameters*	*Island Lesions (n*=*16)*	*Edge Lesions (n*=*15)*
$\left[{\text{MLD}}_{\text{MC}}-{\text{MLD}}_{\text{PB}}\right]\left(\text{Gy}\right)$	0.2±0.2	0.2±0.1
$\left[V_{20,\text{MC}}-V_{20,\text{PB}}\right]\left(\%\right)$	0.5±0.5	0.5±0.3
$\left[D_{1,\text{MC}}-D_{1,\text{PB}}\right]\;\text{of}\;\text{Cord}\;(\text{Gy})$	1.3±1.0	0.7±0.4
$\left[D_{1,\text{MC}}-D_{1,\text{PB}}\right]\;\text{of}\;\text{Esophagus}\;(\text{Gy})$	1.2±1.0	0.9±0.5
$\left[D_{1,\text{MC}}-D_{1,\text{PB}}\right]\;\text{of}\;\text{Brachial}\;\text{Plexus}\;(\text{Gy})$	−0.06±0.10	0.2±1.0

**Figure 9:**
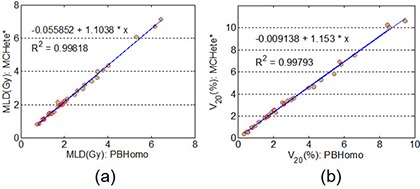
Correlations for the MLD (a) and the $V_{20}$ (b) of lungs for plans calculated with the PB without heterogeneity correction and recalculated with the MC with heterogeneity correction.

Figure 9 shows an excellent correlation on MLD and $V_{20}$ of lungs between PB calculations without heterogeneity correction and MC with heterogeneity correction. The slopes of the linearly fitted lines for MLD and $V_{20}$ were 1.10 and 1.15, respectively. The PB calculation without heterogeneity correction would result in 10% underestimation of MLD and 15% underestimation of the $V_{20}$ of lungs when compared to the MC calculation with heterogeneity correction.

## IV. DISCUSSION

For dose calculations in the thorax region, two competing factors affect dose deposition in the target volume surrounded by the low‐density lung tissue. One is the increased photon fluence due to less attenuation of lung tissue. The other is the lack of electron equilibrium in the interface between air and the solid tumor due to increased range of electrons in the low‐density lung. The increased photon fluence is the dominant effect at the tumor center where all beams intersect. Therefore, the maximum dose in targets with large volumes would be higher if the heterogeneity correction were applied. At tumor edges, both effects interfered with each other. The net effect may depend on the tumor size and location, and may be similar to the situation without heterogeneity correction in certain cases.

The excellent agreement as shown in comparisons between plans PBHomo and MCHomo has demonstrated the equivalence of the MC and PB calculations for homogeneous medium. However, significant differences were observed when comparing MC and PB calculations with heterogeneity correction. Comparisons between plans PBHete and MCHete have shown an average reduction of 23.8%/15.3% in $D_{95}$ of the PTV for island and edge lesions, respectively. A similar discovery for a different lung SBRT dose scheme was reported recently by Chetty.^(^ ^22^ ^)^

The comparisons between PBHomo and MCHete* plans are most interesting. Our results indicated that $D_{95}$ calculated using PB without heterogeneity correction and MC with heterogeneity correction agreed with each other within 1% for edge tumors with volumes larger than 25.1 cc. For island lesions with volumes larger than 22.6 cc, MC calculations showed an average of 3% higher $D_{95}$ than PB calculations. Since $D_{95}$ is more clinically relevant, this comparison demonstrated that simple dose calculation algorithms without heterogeneity correction may approximate calculations using a more sophisticated dose calculation algorithm with heterogeneity correction for edge tumors with large volumes. For small lesions, the MC calculated dose was generally less than PB calculations showing overestimation of dose by using the PB algorithm. For island lesions, PB calculations overestimated $D_{95}$ by 9% for tumor volumes less than 22.6 cc. For edge lesions, PB calculations overestimated $D_{95}$ by 5% for tumors smaller than 25.1 cc. This comparison indicated the demand for more accurate dose calculation algorithms when the tumor volume was small. For both island and edge lesions, a higher hot spot in the PTV expressed by $D_{1}$ was observed in MC calculations with heterogeneity correction regardless of tumor sizes.

In contrast to a recent study reported by Xiao et.al.^(^ ^15^ ^)^ who suggested using 18 Gy instead 20 Gy per fraction for the treatments following RTOG 0236 if superposition convolution type of algorithms are used with the heterogeneity correction, our study indicated that a simple rescaling would not compensate for the differences of applying the heterogeneity correction for all cases. Strong dependence on tumor sizes and locations was observed from this study. For example, the MC calculation for an edged tumor with large volume (greater than 25.1 cc in this study) agreed in 1% with PB calculation without heterogeneity correction. Thus, a rescaling of prescription from 60 Gy to 54 Gy would result in a 10% underdose to the tumor target.

As demonstrated in recalculations of clinical plans with MC and heterogeneity correction, there was more than a 7% dose difference in $D_{95}$ among 38.7% of all cases. Despite underdosing from the Monte Carlo calculations, we observed an excellent control rate. To understand this point, a simple calculation from biologically effective dose (BED) may provide an explanation. Using a α/β of 10 Gy, the BEDs for the two fractionation schemes in this study were already sufficiently high (120 Gy for single fraction of 30 Gy and 180 Gy for 60 Gy in three fractions). As shown in (Fig. 7c1), in 96.7% (30/31) of the patients, a reduction of less than 17% in $D_{95}$ was observed when comparing MC calculations with heterogeneity correction to PB without heterogeneity correction. The 17% reduction in the physical dose from MC calculations with heterogeneity correction still makes BED greater than 100 Gy. According to Guckenberger,^(^ ^23^ ^)^ the dose‐response curve has a plateau near 100 Gy BED. Therefore, we would not expect a significant drop in the local control for this group of patients where the dose was calculated using the PB algorithm without heterogeneity correction.

For plans that were calculated requiring heterogeneity correction, in 93.5% of the patients we observed that the dose difference in $D_{95}$ was more than 7% of the prescription, and an average reduction of 23.8%/15.3% were present in $D_{95}$ for island/edge lesions, respectively, between MC and PB calculations. A possible impact of the reduction of dose for these cases on tumor local control rate is another interesting topic and will be presented elsewhere.

Regarding the dose to OARs, we found small differences in all of the comparisons and no dependence on tumor locations. This is likely due to OARs often being far from the PTV. Noticeable differences were found at low‐dose region for lungs since MC takes into account lateral electron transportation more accurately in low‐density lung tissue. These differences were not significant. We have also shown excellent correlations of MLD and $V_{20}$ of lungs between two different calculations. Thus, a simple rescaling may be applied to the volumes or doses when considering radiation toxicity to the lungs. When comparing the PB to MC calculation with heterogeneity correction, the PB would result in an overestimation of 5% in MLD and 3% in $V_{20}$ of lungs. The PB calculation without heterogeneity correction would result in an underestimation of 10% in MLD and 15% in $V_{20}$ of lungs than the MC calculation with heterogeneity correction.

In the current study, the effect of tumor motion was ignored and setup errors were not taken into account. These two effects added another level of uncertainty to the “true” dose that the tumor received, which was beyond the scope of this paper. However, tumor motion and setup errors may have an effect on mitigating the “hot spot” within the target. Therefore, the difference in “hot spot” between MC calculations with heterogeneity correction and PB without heterogeneity could be reduced. Also, we would like to point out that these observations were only valid for comparisons between PB and MC calculations.

## V. CONCLUSIONS

In this study, using the Monte Carlo algorithm as a gold standard for dose calculation, we compared the plans recalculated with MC to the plans calculated using the pencil beam algorithm in iPlan RT 4.1.2 TPS for lung SBRT cases. Equivalence between the MC and PB calculations for homogeneous medium was demonstrated. Large discrepancies in dose to the PTV were observed between the MC and PB calculations when the heterogeneity correction was applied. Clinical plans that were calculated with the PB without heterogeneity correction were compared to the MC calculation with heterogeneity correction. Differences between these two calculations were dependent on the PTV volume and location. There was often underdosing for small tumors and overdosing for large tumors. Due to high BED for the two fraction schemes (30 Gy in single fraction and 60 Gy in three fractions) analyzed in this study, we did not expect differences in the local control rate, since BED of more than 100 Gy were still maintained in 96.7% (30/31) of patients. In terms of dose to OARs, small and clinically insignificant differences were present for all comparisons. We also established excellent correlations between MC and PB calculations for MLD and $V_{20}$ of lungs. Since only 35 patients were selected for this study, the conclusions were limited. However, this study provided a further understanding of dosimetry in lung SBRT. In particular, we observed strong tumor volume and location dependence in the differences between PB and MC dose calculations. Thus, a rescaling of prescription dose would not compensate for the differences, and an accurate dose calculation algorithm, such as Monte Carlo, was necessary.
